# Artificial tissue: a future surgical approach for reversing erectile dysfunction – correspondence

**DOI:** 10.1097/MS9.0000000000000276

**Published:** 2023-03-27

**Authors:** Ruhul Amin, Bapi R. Sarkar, Prosanta Pal, Amitava Roy, Talha B. Emran

**Affiliations:** a Faculty of Pharmaceutical Science, Assam Down Town University, Panikhaiti, Guwahati, Assam; b Department of Pharmaceutical Technology, University of North Bengal, Darjeeling, West Bengal, India; c Department of Pharmacy, BGC Trust University Bangladesh, Chittagong; d Department of Pharmacy, Faculty of Allied Health Sciences, Daffodil International University, Dhaka, Bangladesh

It has long been recognised that sexual dysfunction in men is rather widespread. Treatments for sexual dysfunction have improved in recent years. Erectile dysfunction (ED), low libido, and dysfunctional ejaculation are all examples of male sexual dysfunction^1^.

Anxiety over how a performance will go is often at the root of a rapid onset of ED. Only radical prostatectomy or other obvious genital tract injuries causes an abrupt loss of male sexual function other than this psychiatric aetiology^2^. Comparatively, men who have ED due to any other reason often report symptoms between the ages of 40 and 70. Scar tissue from previous traumas causes discomfort and dysfunction in some people with Peyronie’s disease^3^.

Replacement of the injured portion of the penis is often achieved by grafting tissues from elsewhere on the patient’s body. However, the immune system is quite adept at rejecting biological materials that are introduced into the body, and even successful grafts may create issues due to variances in tissue types.

The tunica albuginea (TA) is a network of interlaced, wavy collagen fibres (with a little amount of elastin) that provides the scaffolding for the spongy erectile tissues of mammals. When the soft tissues fill with blood, these strands straighten out to make more space while still keeping everything together. As the TA fibres grow to their maximum length, they stretch to provide hardness, functioning like a hydrostatic skeleton to regulate and restrict shape change and to resist external deformation^4^.

After experimenting with various compositions on a balloon model, the researchers were able to develop a synthetic imitation of these fibres. Researchers discovered that the same direction of expansion and stretch as TA was achieved by stretching isotropic polyvinyl alcohol gel and adding crosslinks to maintain the resultant fibres in parallel alignment^5^,^6^. This mimics the soft-to-firm transition seen in erectile tissue.

Chai *et al*.^7^ have developed bionic TA [artificial tunica albuginea (ATA)]. It can stretch and relax in short bursts repeatedly without becoming tired or losing its tensile strength, and it can even survive needle pricks during suturing.

Following this, they used pigs with TA injuries to test the synthetic tissue. After 1 month, the ATA group had decent outcomes, but they were not ideal. After a saline injection, ATA was able to restore normal erectile function in the pigs, despite its inability to replicate the complex structure of real tissue since it could not replace the function of the other tissues also involved, such as blood vessels.

In this phase, they are concerned with repairing a specific tissue in the penis; in the following phase, they either think about the deficiency or they will build an artificial penis. Despite the fact that we still have a way to go before this technology can be utilised by people, the knowledge gained from this study improves our understanding of robust but malleable materials. This biomimetic strategy for design is not confined to the creation of TA tissues; it may be used for a wide variety of different types of structurally important tissues.

This is because, like the TA, most load-bearing soft tissues are characterised by the presence of curled oriented fibres, which give the mechanical qualities necessary for their function. Such tissue is also present in the vascular system, the colon, the eye, the bladder, the tendons, and the heart; thus, a similar method might be used to aid in their regeneration as well.

## Method

This case report has been reported in line with the CAse REport (CARE) guidelines.

## Ethical approval

This article does not require any human/animal subjects to acquire such approval.

## Consent

Not applicable.

## Source of funding

This study received no specific grant from any funding agency in the public, commercial, or not-for-profit sectors.

## Author contribution

R.A.: conceptualization; R.A., B.R.S., B.P. and A.R.: data curation and writing – original draft preparation, reviewing and editing; T.B.E.: writing – reviewing and editing, visualisation and supervision.

## Conflicts of interest disclosure

All authors report no conflicts of interest relevant to this article.

## Research registration unique identifying number (UIN)

None.

## Guarantor

Talha Bin Emran, PhD, Associate Professor, Department of Pharmacy, BGC Trust University Bangladesh, Chittagong 4381, Bangladesh. Tel.: +880 30 335 6193, fax: +880 31 255 0224. https://orcid.org/0000-0003-3188-2272. E-mail address: talhabmb@bgctub.ac.bd

## Provenance and peer review

Not commissioned, externally peer-reviewed.
